# Predictors of help-seeking behaviors in women with urinary incontinence: Based on Iranian women’s lens

**DOI:** 10.1371/journal.pone.0289785

**Published:** 2023-08-04

**Authors:** Fahimeh Rashidi Fakari, Sepideh Hajian, Soodabeh Darvish, Hamid Alavi Majd

**Affiliations:** 1 Department of Midwifery, School of Nursing and Midwifery, Shahid Beheshti University of Medical Sciences, Tehran, Iran; 2 Department of Obstetrics & Gynaecology, Fellowship of Female Pelvic Floor Medicine and Reconstructive Surgery, Shahid Beheshti University of Medical Sciences, Tehran, Iran; 3 Department of Biostatistics, School of Paramedicine, Shahid Beheshti University of Medical Sciences, Tehran, Iran; Queensland University of Technology, AUSTRALIA

## Abstract

Urinary incontinence has an undeniable impact on the quality of life of affected women; however, talking about incontinence is not comfortable for many women, and they often hide it and do not seek treatment. Predictors affecting women’s decisions to seek treatment in communities can be different. This study was designed to identify predictors of help-seeking behaviors among Iranian women with urinary incontinence.

**Methods:**

This cross-sectional, analytical study was conducted on 199 women with urinary incontinence who met the inclusion criteria by convenience sampling from the beginning of 2020 to the middle of 2021. The Incontinence Severity Index, Bradley’s Questionnaire for Urinary Incontinence Diagnosis, Medical Embarrassment Questionnaires, Brief-Illness Perception Questionnaire, Incontinence Quality of Life Questionnaire, Barrier to Incontinence Care Seeking Questionnaire, Medical Help Seeking Scale, and Medical Outcomes Study Social Support Survey were all self-administered data collection tools used in this study. Multiple linear regression was used to investigate the relationship and prediction of help-seeking behaviors by other variables. To analyze the data, SPSS software version 20 was used.

**Results:**

The variables of shame, barriers to care, social support, quality of life, and age were found to be predictors of help-seeking behavior in the research population of women with urinary incontinence. Help-seeking had a direct relationship with quality of life and an inverse relationship with other factors. Among these factors, shame has the greatest impact (P = 0/001, β = - 0/37).

**Conclusions:**

The extracted predictors, especially the variable of "shame" as the most important negative factor related to the treatment decisions of women with urinary incontinence, will help to health service providers to take into account these factors in the regular service provision programs that promote women’s health, which are effective in facilitating the help-seeking of sufferers and correct guidance towards treatment or rehabilitation.

## 1. Introduction

Although the possibility of urinary incontinence (UI (increases with age in women, urinary incontinence is one of the problems of women of various ages, so that the prevalence is estimated up to 70% and the average 25–45% in different countries, with the most studies in Europe and North America [1]. UI is associated with negative physical, psychological, and social consequences [2]. Furthermore, UI affects a woman’s sexual activity, so that affected women may experience various problems of sexual disorders such as desire problems, orgasm, dyspareunia, etc. Also, UI affects sexual satisfaction and frequency of intercourse [3]. UI has also been linked to decreased concentration, physical activity, and job performance [4]. In other words, UI has an undeniable effect on the quality of life of affected women [2].

On the other hand, UI is a social stigma, so talking about it is not easy for affected people [5]. Because the excretion process is one of the private functions of every human being, it is usually kept secret, while the affected people may experience unpleasant conditions from the involuntary excretion of urine [6].

Therefore, many women still use coping strategies to deal with the problem of incontinence. Coping strategies include limiting physical, social, and religious activities, frequent bathing and urinating, washing with hot water, frequently changing their underwear, using pads and drying products, limiting liquid consumption, keeping the body warm, and even resorting to catheter placement [7,8]. on the other, some strategies, such as catheterization and fluid restriction, have side effects and consequences [8]. Also, the cost of using sanitary products is undeniable [9].

Hence, self-management strategies are often a substitute for seeking professional help to control UI, and women’s decision-making for UI treatment is limited, despite therapeutic advances [10–12].

Help-seeking behaviors have been predicted by different factors in societies due to religious and cultural influences on some factors [6,12,13]. In a study in China, perceived social impact and perceived self-efficacy have been introduced as predictors of the intention to seek help for UI in women [13]. While in another study, age, duration of illness, level of education, and previous help-seeking have been reported as variables affecting the willingness to seek help [14]. In another study as well, social support, quality of life, and severity of UI were among the predictor variables [15]. Shame and attitudes toward using health care have also been identified as factors influencing help-seeking behaviors in women with UI [16].

Thus, since the predictors of help-seeking behaviors in women with UI have been reported to be diverse [12,13,15], it is indeed more appropriate to use predictors extracted from the context of every society for health planning and designing interventions, because they are closer to the reality of people’s behavior and more tangible. Caregivers’ awareness of the predictors of help-seeking behaviors, the intensity of the impact, and the main causes will help them better understand the patients and as a result, help them in their initial diagnosis more effectively.

Although, in a recent survey, the overall prevalence of UI in Iranian women was 46% [17], and the effective evidence on the quality of life of these women [18,19], it has been reported that most patients use self-control strategies instead of seeking treatment [20].

## 2. Aim of the study

This study therefore aimed to determine the predictive factors and how they relate to help-seeking behaviors of Iranian women with urinary incontinence.

## 3. Materials and methods

### 3.1. Study design

This cross-sectional, analytical study was conducted from the beginning of 2020 to mid-2021. The research subjects included women with UI who met the inclusion criteria. Sampling was done using the convenience method.

### 3.2. Study population

The inclusion criteria in this study were: being Iranian, being able to read and write, being over 18 years old, not being pregnant, reporting symptoms of UI for at least 6 months, not suffering from a known acute mental illness and not suffering from neuromuscular diseases that disturb the urinary system (such as multiple sclerosis and diabetes mellitus). The exclusion criteria included an unwillingness to continue answering the questionnaires (for any reason). To test the model and according to the valid references that the number of samples is determined based on predictor variables, 20 sample have been suggested for each independent variable, which according to the statistical consultant for the strongest results, the sample size 200 were considered [21–23].

### 3.3. Study settings

The research environment was the gynecology clinics of two teaching hospitals with a different location in terms of socioeconomics, in order to maintain variation (With greater cooperation of the officials of these centers and the high number of clients). Furthermore, for maximum variations of the samples, according to the opinion of the research team, it was preferred to take samples from private centers as well. Therefore, according to the research sources, an obstetrician’s office in Tehran, that was willing to voluntarily cooperate with the study was also used. The subjects were selected among the women who were referred to those centers for the treatment of their gynecological diseases.

It should be noted that the selection of gynecology clinics and midwifery offices as the research environment was to make it easier for the patients to respond in an environment where only female patients were present (considering the sensitivity of the subject for patients).

### 3.4. Data collection

At the beginning of the sampling, Bradley’s Questionnaire for Urinary Incontinence Diagnosis (QUID) was used to confirm the diagnosis of UI and also to diagnose the type of incontinence (stress, urge, or mixed) in the participants by researcher. This questionnaire contains six items, and the answers are scored from 0 to 5, from never to always. For stress incontinence, the answers of items 1, 2, and 3 are added together, and for urge incontinence, the answers of items 4, 5, and 6 are added together. The cut-off score for stress incontinence is 4 or more, and the cut-off score for urge incontinence is 6 or more. If a person is diagnosed with both types of incontinence, meaning that the total score in the stress section is 4 or more and the total score in the urgency section is 6 or more, the patient is diagnosed with mixed urinary incontinence [24]. QUID has also been psychometrically evaluated in Iran, and the calculation of Cronbach’s alpha coefficient (0.9) and Intra-class Correlation Coefficient (ICC) (0.86) indicate that its reliability is appropriate [25]. The ICC for internal homogeneity in the current study ranged between 0.75 and 0.91.

In addition to the QUID, data collection tool was a questionnaire of demographic characteristics and characteristics related to UI, as well as standard questionnaires according to the variables extracted from the experience of Iranian women with UI regarding help-seeking behaviors (which includes perception of disease, shame, social support, quality of life, and barriers to the care system) [26], which were completed by the participants through the self-completion method.

Brief-Illness Perception Questionnaire was used to access the participants’ understanding of UI [27]. For this reason, Brief-Illness Perception Questionnaire, was used which included dimensions and questions that showed knowledge and understanding of the disease the cover the variable of perception. This questionnaire was also psychometrically evaluated in Iran by Bazazian and Besharat in 2010 [28]. It should be noted that the reliability of the perception questionnaire in this study was calculated using the method of stability (intra-class correlation coefficient) of 0.95.

The variable of quality of life was measured by Incontinence Quality of Life (I-QOL) which has 22 items and 3 subscales. The responses to the items are on a 5-point Likert scale, and a higher score indicates a better quality of life [29]. This questionnaire has also been psychometrically evaluated in Iran, and the validity of its 3-aspect structure has been confirmed [30]. In the present study, Cronbach’s alpha coefficient was 0.95 and the ICC value was 0.98.

The Barrier to Incontinence Care Seeking Questionnaire (BICS-Q) consists of 14 items that were used to investigate the variables of care system barriers such as cost and so on. The range of responses to this questionnaire is on a 4-point Likert scale from zero (not at all) to three (greatly). In this questionnaire, a higher score means that there are more barriers to seeking incontinence care, and vice versa [31]. In this study, the Cronbach’s alpha coefficient of the BICS-Q was calculated to be 0.63 for internal consistency and 0.74 for stability.

The assessment of the shame variable was done by the Medical Embarrassment Questionnaire (MEQ), which has two sections: bodily embarrassment and judgment concern. The scoring on this scale is from 1 (not at all or never) to 5 (very much or always) [32]. Moreover, for the variable of social support, the Medical Outcomes Study-Social Support Survey (MOS-SSS), which was designed by Sherbourn and Stewart, was used, and the range of responses was from none of the time (1 score) to all of the time (5 score) [33]. It should be noted that in this study, according to the opinions of 15 experts in the fields of instrument making and medicine, some questions of the MEQ and MOS-SSS scales were used, and validity and reliability were also carried out. The minimum value of the content validity ratio in among the questions of the MEQ and MOS-SSS scales was 0.6, that this value is acceptable based on the Lawshe’s table [34]. Also, the minimum value of the content validity index for their questions was more than 0.79, which is considered appropriate [35]. In addition, the internal consistency for the questions of the MEQ was 0.89 and the stability value was 0.94, and for the MOS-SSS, it was 0.83 and 0.96, respectively.

The investigation of the help-seeking variable, the main variable of the research, was done by the Medical Help Seeking Scale, which includes 12 questions and 2 subscales. It has been psychometrically evaluated as a separate instrument by DiLorenzo et al. (2015). The scoring answers for straight questions is 3-2-1-0 and for reverse questions 0-1-2-3 and for the responses to the items are on agree, partly agree, partly disagree, and disagree [36]. This tool has also been psychometrically tested in Iran which has been confirmed by the construct validity of two subscales, and its reliability has been obtained by test-retest (0.85) and split half (0.78) method [37]. In this study, the internal consistency was 0.82 and the stability value was 0.89.

The Incontinence Severity Index, developed by Sandvik et al., was used to examine the individual variable of disease severity. In this scale, the severity of incontinence is measured based on the amount of urination and the frequency of urination and includes 2 questions, whose scores are multiplied to get the overall score of incontinence severity [38,39]. In this study, the ICC was calculated at 0.78.

It should be noted that in the selection of questionnaires, with the supervision and consultation of the research team, as much as possible the following criteria were considered to select appropriate questionnaires for the variables: having items covered that are stated about each predictor variable in the related studies, medical questionnaires, specific to urinary incontinence, considering the number of questions, previously psychometrics in this community.

### 3.5 Data analysis

After collecting, transferring, and coding the study data, they were analyzed using SPSS version 20 software. Descriptive statistics such as mean, standard deviation, and frequency distribution were used to investigate the characteristics of the participants in the research.

Multiple linear regression was used to investigate the relationship and prediction of help-seeking behaviors by other variables. As a result, the help-seeking variable was considered a dependent variable, and the background variables (perception of the disease, shame, social support, quality of life, barriers to the care system) were entered into the model as independent variables, along with individual variables (age, duration, severity, types of incontinence). The P value in this research is considered to be less than 0.05.

### 3.6 Ethics

Ethical considerations were taken into account in this study through permission from the university vice chancellor, obtaining a code of ethics from the university’s ethics committee (IR.SBMU.PHNM. 1397.033), and obtaining written informed consent from the participants. Adequate explanations about the optionality of participation, the confidentiality of authors access to information that could identify individual participants during and after, and the possibility of withdrawing from the study at each stage were provided to the participants.

## 4. Results

Two hundred women with UI were eligible to enter the study. One questionnaire was discarded due to missing data; therefore, analyses were performed on the data of 199 participants. The average age of the participants was 46.52 ± 12.20 and their least age was 25 and their most was 87 years. The average parity of the women was 2.5 ± 1.7, the minimum was 0, and the maximum was 10. The average duration of incontinence was 5.6 ± 3.8 years, and the minimum and maximum duration were 1 and 20 years, respectively. Other demographic characteristics related to UI are reported in Table 1.

**Table 1:** Characteristics of participating.

Characteristics	199 (%)
**Education**	
Illiterate	8(4.0)
Primary school	38(19.1)
Secondary school	29(14.6)
High school	14(7.0)
Diploma	36(18.1)
Higher than diploma	74(37.2)
**Occupation**	
Housewife	131(65.8)
Employee	63(31.7)
Retired	5(2.5)
**Income** (Individual evaluation of the economic situation)	
Less than subsistence	37(18.6)
To the extent of subsistence	153(76.9)
More than subsistence	9(4.5)
**Type of urinary incontinence**	
Stress	76(38.2)
Urge	46(23.1)
Mixed	77(38.7)
* **Intensity**	
Mild (1–2)	4(2.0)
Moderate (3–6)	87(43.7)
Severe (8–9)	80(40.2)
Very severe (12)	28(14.1)
**Duration (years)**	
2 ≥	46(23.1)
3–5	76(38.2)
6–10	63(31.7)
10<	14(7.0)
**Help-seeking behaviors previous**	
No	177(88.9)
Yes	22 (11.1)

* Based on Incontinence Severity Index.

To start the analysis, the relationships between the independent variables and the main variable were checked through univariate tests (Table 2), so that the relationships with the strongest potential were included in the model. According to the obtained findings, the variables of disease severity, perception of the disease, and types of incontinence were not included in the model due to the lack of a strong relationship with the dependent variable. Finally, the variables of shame, social support, barriers to care, quality of life, age, and duration of illness were entered into the multiple regression model as independent variables and help-seeking as a dependent variable, and the analysis was performed using the Enter Method.

**Table 2:** Relationships between the predictor variables and the help-seeking before entering the model.

Variables	P. Value
Age	0.003
Severity of Incontinence	0.84
Duration	0.003
Urge vs. Stress	0.23
Mixed vs. Stress	0.82
Perception of the Disease	0.31
Shame	0.001
Social Support	0.001
Barriers to Care	0.005
Quality of Life	0.02

One of the assumptions of multiple regression is the absence of collinearity between independent variables. In this study, the tolerance index (TI) and variance inflation factor (VIF) were used to detect collinearity. The values of the tolerance index in the two variables of age and disease duration were close to the level of 0.4, which is because at this level the probability of collinearity increases [40], so it was preferred to remove the disease duration variable from the model, which improves tolerance and variance inflation. As a result, for the shame variables (TI = 0.92, VIF = 1.08), social support (TI = 0.87, VIF = 1.14), care barriers (TI = 0.90, VIF = 1.10), quality of life (TI = 0.96, VIF = 1.04), and age (TI = 0.92, VIF = 1.07) were obtained.

Another assumption of multiple regression is to check for the absence of autocorrelation in the residuals of the regression model, which was done by the Durbin-Watson test, and in this study, the value (Durbin-Watson statistics = 1.630) was suitable. Also, the normal distribution of the residuals was checked and confirmed by the Kolmogorov-Smirnov test (P = 0.20) before calculating Durbin-Watson.

**Fig 1:**
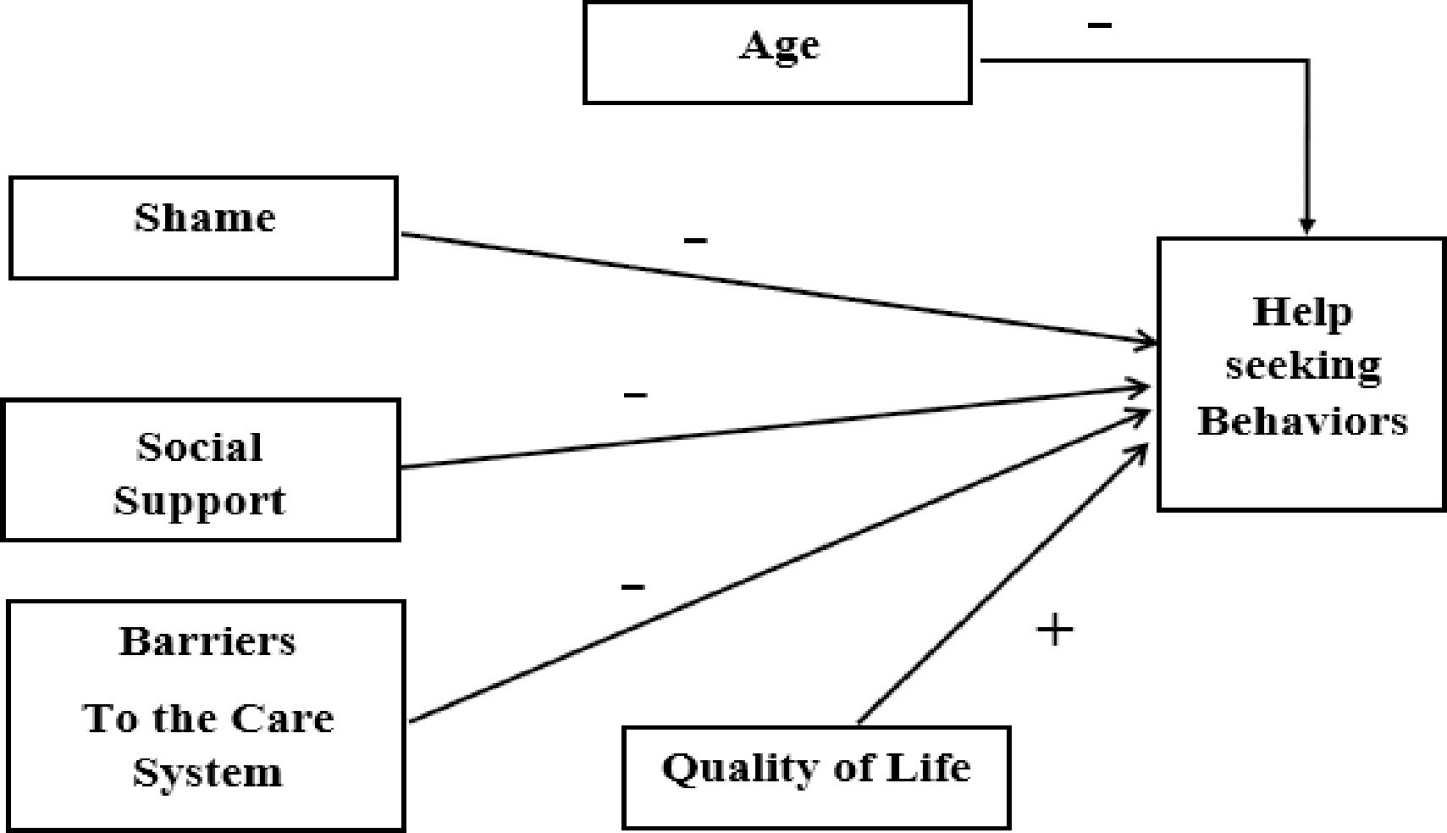
Schematic view of predictors.

The relationship between predictors and seeking treatment can be seen in Table 3. Thus, an inverse relationship was found between the four variables of shame, care barriers, social support, and age with help seeking; in other words, as they increase, help seeking decreases (and vice versa); also, a direct relationship was observed between the quality of life variable and help seeking. In addition, in the ranking of the intensity of the effect of the variables on help-seeking, based on the comparison of the standard estimates, the variable of shame ranked first and had the most effect (β = -0.37). As a result, for every unit increase in shame, help-seeking decreases by 0.24 units on average, assuming all other independent variables remain constant. Schematic view of predictors of help-seeking behaviors is drawn in Fig 1.

**Table 3:** The relationship between predictors and seeking treatment.

	B Unstandardized Coefficients	Standard Error	Beta Standardized Coefficients	P. Value
**Shame**	-0.243	0.043	- 0.374	0.001
**Social support**	-0.162	0.055	-0.198	0.004
**Barriers to the care system**	-0.102	0.102	-0.066	0.320
**Quality of life**	0.024	0.023	0.067	0.295
**Age**	-0.058	0.035	-0.107	0.101

## 5. Discussion

The results of the study confirmed the role of the variables of shame, barriers to care, social support, quality of life, and age as predictors of help-seeking behavior in Iranian women with UI. Furthermore, there was a direct relationship between help-seeking and the quality of life variable, and an inverse relationship was observed with other factors. According to the findings of this study, shame appears to be a significant factor in women seeking help for UI treatment in this society, as it has taken the first place among the influencing factors in the model, and the inverse relationship with help-seeking confirms its deterrent role. Various studies have produced contradictory findings regarding the effect of shame on seeking treatment. For instance, Dugan et al. did not report shame as an influencing factor in the decision to seek help [41]. While Biswas et al. stated that shame is the cause of almost 56 percent of the reasons for not seeking treatment [42]. The inhibiting role of shame may relate to feeling judged (by others or caregivers) or physical shame (from examination, especially by a male caregiver). As a result of the shame of the sense of judgment, the woman avoids going to diagnostic and treatment centers for incontinence and also do not express the problem of UI to caregivers, which appears to be related to societal beliefs. As well as physical shame, which makes a woman avoid seeking help due to the probability of examination, especially by a non-same-sex caregiver. Physical shame may be due to religious values and cultural attitudes, despite the fact that there are few female urologists in this society. As a result, shame was to be expected. In addition, gender assumption of the disease may also be one of the reasons for some women’s reluctance to visit a male doctor, as Hadar Amir et al. reported that a sense of comfort could be one of the reasons why women prefer to choose a female doctor [43]. It may be due to the hypothesis of a better understanding of disease problems with common female anatomy and physiology. However, considering that shame has the most importance in the behavior of seeking help for treatment in the present study, the importance of the role of health workers, especially for the diagnosis of women with UI, as well as the necessity of consultation and referral to specialized centers based on the preferences of patients, is shown to be greater than before.

Barriers to care were another predictor obtained in this study, which showed an inverse relationship with seeking treatment. So, with the increase in barriers to care, the probability of seeking help will decrease. In other words, the more the patient is directly or indirectly faced with the weak and unsatisfactory structure of service provision for diagnosis and treatment, the stronger the obstacle to seeking treatment, which reduces the probability of seeking help later. Barriers to care can be a variety of problems in the health system, including inconvenience, cost, and relationships, which have been reported in studies and affect people’s willingness to attend and receive services [31,44,45]. Improving service delivery leads to increased satisfaction and as a result higher attendance; in fact, by removing the barriers of the care system, it becomes easier to seek help; and vice versa, in poor quality care, the number of referrals and even follow-ups are reduced, forcing patients to use self-management strategies. Furthermore, insurance companies’ weak commitments to cover the costs of diagnosis and treatment, as well as the increasing amount of out-of-pocket payment, exacerbate care problems.

Regarding the variable of social support, contrary to expectations, the result of the study showed that patients with more social support were less likely to seek help. In other words, when all forms of social support, including emotional, material, and so on, are reduced from family and friends, the likelihood of seeking help and following through on treatment increases. Kang et al. reported that more social support in patients was a facilitating factor for UI help-seeking [15], which is not consistent with the present study. One of the probable reasons for this finding may be related to patients’ fear of dependence and feeling the need for support from others in the future. As people with poor social support (compared to people who are encouraged by the support of others) have a sense of loneliness and worry about their condition worsening with the progress of the disease and aging, a greater perception of risk is created in them, and they may seek more help. Therefore, the emergence of such in the inverse relationship between support and help-seeking is not far from expected. On the other hand, the majority of the current study’s participants had an academic education, which has an impact on the ability to make a decision to seek help and is not without influence in the occurrence of a different result regarding the discussed variable.

The results of the present study showed a positive estimate between the predictor variable, quality of life, and the help-seeking behavior. Kang et al. reported an inverse relationship between the quality of life score and help-seeking [15], which is contradictory to the present study. The explanation of the emergence of a positive relationship between quality of life and help-seeking in the current study can be related to the smaller number of participants with disabling (very severe) incontinence. In other words, if the severity of the symptoms is insufficient to significantly alter one’s quality of life, and if other factors related to help-seeking discussed in this study (including shame, which was the strongest predictor of help-seeking), the number of people seeking help will not change significantly. In addition, the quality of life undergoes changes from the beginning of the onset of symptoms, but at first, the person tries to manage the symptoms by herself until the severity of the disease increases to such an extent that strategies cannot help to control the symptoms of the disease. Therefore, it seems that the severity of the symptoms and signs of the disease in the participants was not severe enough to change the attitude of the patients to seek help. Pakgohar et al. also reported that there was no relationship between patients’ quality of life and help-seeking which is consistent to the present study. It should be noted that in their study, also, the number of participants with severe incontinence was not numerous [46].

Also, in this study, a negative relationship between the age variable and help-seeking was obtained, so that with increasing age, help-seeking decreases. There is a contradiction regarding the age variable, so that some studies have introduced increasing age as a factor for seeking help, while others, like the present study, reported the opposite [14,47]. One of the probable causes of the current finding is the multitude of problems during old age and prioritizing other problems, as well as adaptation with the disease due to a misunderstanding of the lack of treatment for the disease or concern about surgery in old age, which can be one of the reasons for distancing from seeking help for incontinence. In addition, UI for elderly women may be perceived as a normal or common condition, and for this reason they do not seek help. Normalization with increasing age has also been observed in the experiences of women with UI in this community [26]. Older women usually attribute the disease to a natural process caused by aging, especially because they may share experiences with their peers who suffer from this condition. Therefore, the stigma caused by the disease is felt less at this age compared to young women. In other words, the reduction of help-seeking with increasing age can be related to the reduction of the stigma of the disease in old age, so that the stigma caused by this disease is much greater for younger women, so it is more likely to draw them to medical centers. On the other hand, the effect of the decrease in sexual attractiveness in old age on seeking help should not be neglected. So that UI affects sexual relations, and because matters related to sexual function have a lower priority for women as they age, and since there is less effort to maintain sexual relationships in line with the goal of stabilizing married life during this period, older women are less encouraged to seek treatment for incontinence [3,48,49].

### 5.1. Strengths and limitations

Among the strengths of the present study, our knowledge is that, for the first time, the predictors of help-seeking behaviors of women with UI were investigated based on the information obtained from the experiences of women in this society; therefore, extractive factors can be more tangible with patients’ behavior and decisions, and making the caregivers aware of those factors will help them better understand the patients, and as a result, an early diagnosis will help more effectively. In addition, emphasizing diseases that receive less attention in a society is an important step in attracting the attention of health policymakers and can lead to the reduction of barriers to care.

One of the limitations of this research is that it relies on the opinions of the participants, who were conveniently selected, which is one of the limitations of studies that use paper tools. However, at the beginning, the importance of the research and the necessity of accurate answers were clearly emphasized to the participants.

## 6 Conclusions

The results of the study confirmed the variables of shame, barriers to care, social support, quality of life, and age as predictors of help-seeking behavior in Iranian women with UI, and the shame variable was found to be the most important factor related to the treatment decision of women with UI. Extracting predictive factors from the context of society as well as knowing the degree of importance and intensity of these influencing factors is a suitable guide to being more realistic in planning for health interventions and allocating resources in order to help reduce the perceived obstacles. Also, knowing the factors will help the health service providers take into account these factors in the regular service provision programs that promote women’s health, which are effective in facilitating the help-seeking of sufferers and correct guidance towards treatment or rehabilitation, so that affected women can experience a better quality of life.

## Supporting information

S1 Checklist STROBE statement—Checklist of items that should be included in reports of observational studies. (DOCX)
